# Immunization of mice by Hollow Mesoporous Silica Nanoparticles as carriers of Porcine Circovirus Type 2 ORF2 Protein

**DOI:** 10.1186/1743-422X-9-108

**Published:** 2012-06-12

**Authors:** Hui-Chen Guo, Xiao-Ming Feng, Shi-Qi Sun, Yan-Quan Wei, De-Hui Sun, Xiang-Tao Liu, Zai-Xin Liu, Jian-Xiong Luo, Hong Yin

**Affiliations:** 1 State Key Laboratory of Veterinary Etiological Biology and National Foot and Mouth Disease Reference Laboratory, Lanzhou Veterinary Research Institute, Chinese Academy of Agricultural Sciences, Xujiaping1, Lanzhou, Gansu, 730046, The People’s Republic of China

**Keywords:** Hollow mesoporous silica nanoparticles (HMSNs), Porcine circovirus type 2 (PCV2): ORF2, Delivery, Immunization, Mice

## Abstract

**Backgroud:**

Porcine circovirus type 2 (PCV2) is a primary etiological agent of post-weaning multi-systemic wasting syndrome (PMWS), which is a disease of increasing importance to the pig industry worldwide. Hollow mesoporous silica nanoparticles (HMSNs) have gained increasing interest for use in vaccines.

**Methods:**

To study the potential of HMSNs for use as a protein delivery system or vaccine carriers. HMSNs were synthesized by a sol–gel/emulsion(oil-in-water/ethanol) method, purified PCV2 GST-ORF2-E protein was loaded into HMSNs, and the resulting HMSN/protein mixture was injected into mice. The uptake and release profiles of protein by HMSNs *in vitro* were investigated. PCV2 GST-ORF2-E specific antibodies and secretion of IFN-γ were detected by enzyme-linked immunosorbent assays, spleen lymphocyte proliferation was measured by the MTS method, and the percentage of CD4+ and CD8+ were determined by flow cytometry.

**Results:**

HMSNs were found to yield better binding capacities and delivery profiles of proteins; the specific immune response induced by PCV2 GST-ORF2-E was maintained for a relatively long period of time after immunization with the HMSN/protein complex.

**Conclusion:**

The findings suggest that HMSNs are good protein carriers and have high potential for use in future applications in therapeutic drug delivery.

## Background

Clinical and laboratory studies have shown that porcine circovirus type 2 (PCV2) is a primary etiological agent of post-weaning multi-systemic wasting syndrome (PMWS). PMWS is clinically characterized by anemia, jaundice, severe weight loss, and histopathological lesions, including lymphocyte depletion and infiltration of monocytes in lymphoid tissues. Morbidity and mortality with PMWS are severe in acute outbreaks, usually resulting in the death of 70% to 80% of affected animals [1,2]. Hence, PMWS is a disease of increasing importance to the pig industry worldwide, and determination of methods with which to protect the piglets from PCV2 infection is a current research hotspot. Immunization against PCV2 has been studied intensively and found to be the most effective strategy for protecting pigs against PCV2 infection.

PCV2 contains a single-stranded circular DNA genome of about 1.76 Kb, having three large open reading frames (ORFs) [3-5], namely, ORF1, ORF2 and ORF3. Capsid protein (Cap protein), encoded by ORF2 of PCV2, which is the major structural protein of the virus with a molecular weight of 27.8 kDa, is the major immunogenic protein and has type-specific epitopes[4,6,7]. Neutralization of monoclonal antibodies [8,9]and swine sera [10]have been shown to react with Cap protein. Therefore, Cap protein has been used as a PCV2 gene for recombinant vaccines[11-13]. However, almost all vaccines prepared by ORF2 proteins expressed in eukaryotic or prokaryotic systems utilize the procedure of primary vaccination followed by boost injectionin order to induce persistent immune responses [11,13-16].To optimize the PCV2 protein vaccine and induce higher and more persistent immune responses, researchers have focused on developing safe and efficient drug delivery vehicles. Since the use of drug delivery by means of controlled technologies began in the 1970s, it has continued to expand rapidly, so much so that there are now numerous products for drug delivery both in the market and in development, including dendrimers, micelles, liposomes, microbubbles, as well as various nanovehicles, including nanoparticles [17,18]. Of these vehicles, hollow mesoporous silica nanoparticles (HMSNs) for biomedical purposes, including drug delivery, have gained increasing interest for use in vaccines. HMSNs have unique structural features, including large surface areas, tunable pore sizes, and well-defined surface properties; these properties indicate that they can be used as carriers for therapeutic compounds in vitro and in vivo. In addition, HMSNs have been approved by the Food and Drug Administration as a new biocompatible material. HMSNs show multifunctional surface modification, controlled release capability, and good thermal stability. Thus, they are ideal nonviral carriers for gene/drug delivery [19,20].

To obtain specific immune responses against PCV2 ORF2 protein, the antigenitic epitope at amino acid residues 113–147 of PCV2 ORF2 [21] was expressed; this epitope was found to be the immunorelevant epitope for virus type discrimination[8]and named ORF2-E. To induce persistent immune responses of PCV2, purified PCV2 GST-ORF2-E proteins were loaded into HMSNs, which were synthesized by a sol–gel/emulsion (oil-in-water/ethanol) method [22] and used as a vehicle for protein delivery with controlled release kinetics. The resulting PCV2 GST-ORF2-E protein-loaded HMSNs were injected into BALB/c mice. The immune responses of mice were then evaluated. Compared with immune responses obtained from using the PCV2 GST-ORF2-E protein, PCV2 GST-ORF2-E protein-loaded HMSNs induced higher humoral and cellular immune responses. The results are very encouraging and demonstrate that HMSNs as a protein delivery vehicle may be further investigated for the development of subunit vaccines based on recombinant proteins.

## Materials and methods

### Synthesis and characterization of HMSNs

Unless otherwise stated, chemicals were obtained from Sigma–Aldrich. The HMSNs were synthesized by a sol–gel/emulsion method with little modification[22]. Briefly, ethanol and H_2_O and tetraethoxysilane (TEOS) and hexadecyltrimethy-lammonium bromide (CTAB) were mixed and continuously stirred. Then, 25% ammonium hydroxide solution (NH_4_OH in H_2_O) was added, and the mixture was stirred for another 3 h to 4 h at room temperature. Following washing with several times deionized water and centrifugation at 8000 rpm to 10000 rpm for 10 min to 15 min, the resulting powders were calcined in air at 200°C for 2 h then at 600°C for 6 h.

Transmission electron microscopy (TEM) and scanning electron microscopy (SEM) were used to determine the morphology and size of the HMSNs. Samples for TEM measurements were prepared by dipping a drop of the colloidal solution onto Formvar-coated copper grids and observed with a JEOL (2001) electron microscope operating at an acceleration voltage of 200 kV. SEM images were taken on a Shimadzu SSX-550 field emission scanning electron microscope at 15.0 kV.

### Expression of protein

PCV2 ORF2-E protein was expressed in *E. coli* BL21 as described previously [21] The GST-ORF2-E fusion protein was purified by a MagneGST™ Protein Purification System (Promega, USA). The GST fusion protein was analyzed by SDS-PAGE and Western blot.

### The size distribution of the HMSN/protein mixture

The size distributions of HMSNs were determined using a Malvern Instruments (Malvern Instruments Ltd., UK) Zetasizer Nano ZS series system (ZEN 3600). Samples of the HMSN/protein complex (1 mg/150 ug; w/w) and HMSNs were suspended (1 mg/mL) in phosphate buffer saline (PBS, pH 7.0). The size of the nanoparticles was calculated using Dispersion Technology Software, version 4.20 (Malvern Instruments Ltd.).

### Protein adsorption of HMSNs

To load the protein into HMSNs, PBS (pH 7.0) solutions containing different concentrations of HMSNs (1, 5, and 10 mg/mL) were sonicated for 15 min, and then mixed with 200 μL of PCV2 GST-ORF2-E protein (2.4 mg/mL in PBS) at room temperature. At different time points, the solutions were centrifuged at 10000 rpm for 5 min, and the amounts of proteins in the supernatants were measured by a Micro BCA^TM^ protein assay kit (Pierce, Rockford, IL, USA) by measuring their UV absorbance at 562 nm. The amount of protein adsorbed onto the silica was estimated by subtracting the protein dissolved in the solution from the amount of protein loaded.

### Release kinetics of HMSNs

HMSNs loaded with PCV2 GST-ORF2-E protein were suspended in 15 mL PBS (pH 7.0). The solution was divided into 15 microfuge tubes (1 mL/tube). The tubes were kept in 37°C for different lengths of time. At certain time points, the solution was centrifuged at 10000 rpm for 5 min. The supernatant containing proteins released by the HMSNs was measured by a Micro BCA^TM^ protein assay kit (Pierce,USA). The amount of protein released by the HMSNs was estimated from the amount of protein in the supernatant.

### Vaccination

All animals received humane care in compliance with the guidelines of the Animal Research Ethics Board of Lanzhou Veterinary Research Institute, CAAS, China. BALB/c mice were purchased from the animal house of Lanzhou Veterinary Research Institute and raised in isolation cages.

Twenty-seven healthy eight-week-old female BALB/c mice were randomized into three groups. The mice in group A were immunized with PCV2 GST-ORF2-E protein-loaded HMSNs, those in group B were immunized with PCV2 GST-ORF2-E protein, and those in group C were immunized with the empty HMSNs in PBS. Every mouse was injected intramuscularly with 100 μg (0.7 mg HMSNs loaded with 100 μg protein) protein in PBS solution using a needle and syringe. Serum samples were collected from the retro-orbital plexus every week after immunization and used in serological tests.

### Immunofluorescence assay

PCV2 infection of PK-15 cells was performed as described previously [21]. Cells were fixed with 4% polyformaldehyde in PBS at room temperature for 30 min and washed with PBST (PBS containing 0.1% Tween20, pH 7.4). The cells were then incubated for 10 min at room temperature with 0.1% Triton X-100 in PBS, followed by incubation for another hour at 37°C with mouse serum diluted 50 times in PBST containing 5% foetal bovine serum (FBS). After three washes with PBST, cells were stained for 1 h at 37°C with FITC-conjugated rabbit anti-mouse IgG (Dako, Denmark) diluted 100 times in PBST containing 5% FBS. After washing, plates were examined by fluorescence microscopy.

### Enzyme-linked immunosorbent assay

Serum samples were collected from mice at intervals of one week and evaluated by an indirect enzyme-linked immunosorbent assay (ELISA) using the recombinant GST-ORF2-E protein of PCV2 as an antigen. The detailed protocol was followed as described [21] with minor modifications. Briefly, 96-well microtiter plates (Nunc, USA) were coated with the recombinant GST-ORF2-E protein of PCV2 in 0.1 M carbonate/bicarbonate buffer (pH 9.6) and incubated overnight at 4°C. After three washes in PBST, the plates were blocked with100 μL PBST containing 5% non-fat dry milk for 1 h at 37°C. After three washes in PBST, diluted mouse serum (1:100) with PBS containing 1% non-fat dry milk was added, and plates were again incubated for 1 h at 37°C. After three washes in PBST, 100 μL diluted rabbit anti-mouse IgG peroxidase conjugate (Sigma,UK) in PBST containing 1% non-fat dry milk at a 1:2000 dilution was then added for 1 h at 37°C. The plates were then washed three times, and the colorimetric reaction was developed using 50 μL substrate solution (FAST o-phenylenediamine dihydrochloride, Sigma) for 15 min at 37°C. Color development was stopped with 50 μL of 2 N H_2_SO_4_, and optical density (OD) was read at 490 nm.

### T-lymphocyte proliferation assay

T-lymphocyte proliferation assay was performed using the Cell Titer 96AQueous Non-Radioactive Cell Proliferation Assay (Promega, USA). Mice spleens were removed in sterile conditions and ground through a sterile cuprous mesh (200 meshes). The spleen cells were immersed in RPMI 1640 medium with 10% FBS, added to lymphocyte separation medium (Sangon, China), homogenized, and centrifuged at 1000 rpm × g for 10 min. Pellets were discarded and buoyant cells were washed three times in RPMI 1640 medium with 10% FBS. T-lymphocytes in 96-well plates (5 × 10^4^ cells per well) were co-cultured with PCV2 GST-ORF2-E protein (2 μg/mL) in RPMI 1640 supplemented with 10% fetal bovine serum (Gibco, Life Technologies, Vienna, Austria), and maintained at 37°C in a humidified 5% CO_2_ atmosphere for 60 h. MTS(3-(4,5-dimethylthiazol-2yl)-5-(3-carboxymethoxyphenyl)-2-(4-sulfophenyl)-2 H-tetra zolium, inner sath) was added to each well, and then incubated for 4 h at 37°C under 5% CO_2_. The absorbance at 490 nm was measured. Results were expressed as a percentage of untreated controls.

### Flow cytometry analysis

To determine the phenotype of the T-cell subpopulation in spleen lymphocytes by flow cytometry, single-labeling methods were employed for defining different subpopulations. Splenocytes (10^6^ cells) were washed in cold PBS containing 1% albumin from bovine serum, centrifuged, and resuspended in cold PBS. The splenocytes were then stained with rabbit anti-mouse CD4: APC/CD8: PE (BD, USA). Cells were incubated for 30 min at 4°C and washed three times with cold PBS buffer. Samples were analyzed using a FACScan system (BD Biosciences).

### Quantification of mouse IFN-γ

A mouse IFN-γ-precoated ELISA kit (Dakewei, China) was used to determine IFN-γ in mouse sera according to the manufacturer’s instructions. The serum was diluted at a ratio of 1:50 and 100 μL of the resulting solution was added to each well. Measurements were done in duplicate and the plate was read immediately at 450 nm on a Universal Microplate Reader (Bio-Rad Instruments, USA). A standard curve for IFN-γ was obtained using the standard protein provided by the manufacturer.

### Statistical analysis

The data are presented as mean ± SD. The statistical analysis was performed using the SAS9.1 statistical software package. First, the verification of the homogeneity of variance by using Levene test was performed. Then, analysis of variance between groups by using One-way ANOVA was applied. Finally, comparison of mean pair wise differences between groups using Least Significance Difference (LSD) was performed. Significance of all statistical tests was set at 0.05 (p < 0.05).

## Results

### Characterization of HMSNs

**Figure 1:**
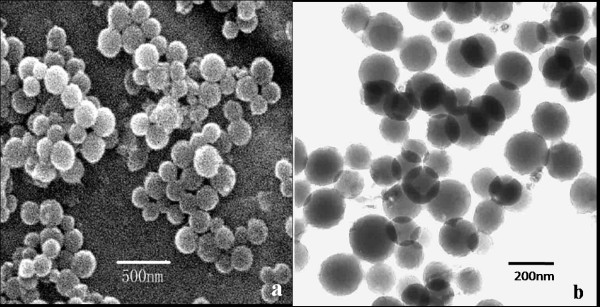
**The morphologies and microstructures of HMSNs.** The HMSNs dispersed in phosphate buffer solution and observed by SEM (**a**) and TEM (**b**).

Hollow mesoporous silica spheres were synthesized by a sol–gel/emulsion (oil-in-water/ethanol) approach, in which cetyltrimethylammonium bromide surfactant was employed to stabilize and direct the hydrolysis of oil droplets of tetraethoxysilane. Figure 1 shows that the resulting particles are spherical shape. SEM images reveal that the spheres have a rough surface and retain their intact spherical nature even after calcination at 600°C for 6 h. TEM and SEM results indicate that the spheres are hollow in character and have an average diameter of about 200 nm (Figure 1a and Figure 1b).

### Size distribution of HMSNs and HMSN/protein complex

**Figure 2:**
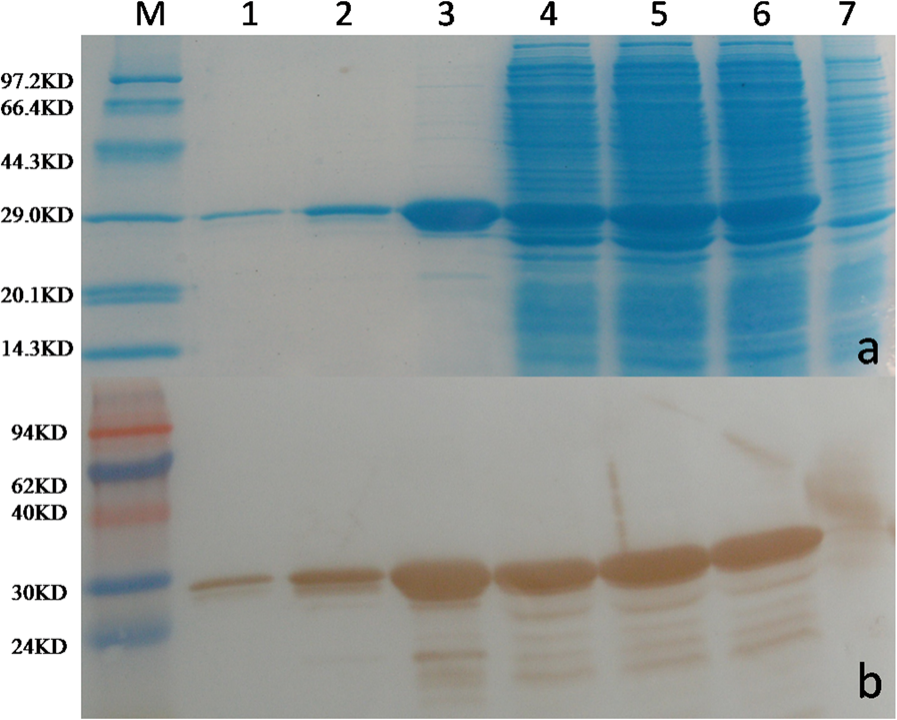
**The expression of GST-ORF2-E protein.** GST-ORF2-E protein was analyzed by (a) SDS-PAGE and (b) Western blot with an anti-GST monoclonal antibody. Lane 1: the third elution; Lane 2: the second elution; Lane 3: the first elution; Lane 4, supernatant of cell lysate after sonication; Lane 5: cell pellet after sonication; Lane 6: BL21 cells lysate after induction of IPTG; Lane 7, BL21 cells lysate before induction of IPTG. A clear band of 29 kDa was observed after induction.

SDS-PAGE and Western blots were used to confirm the expression of the recombinant protein. Figure 2 shows a specific band of about 29 kDa on the SDS-PAGE gel and Western blot membrane when the purified protein was tested (lanes 1 to 3).

**Figure 3:**
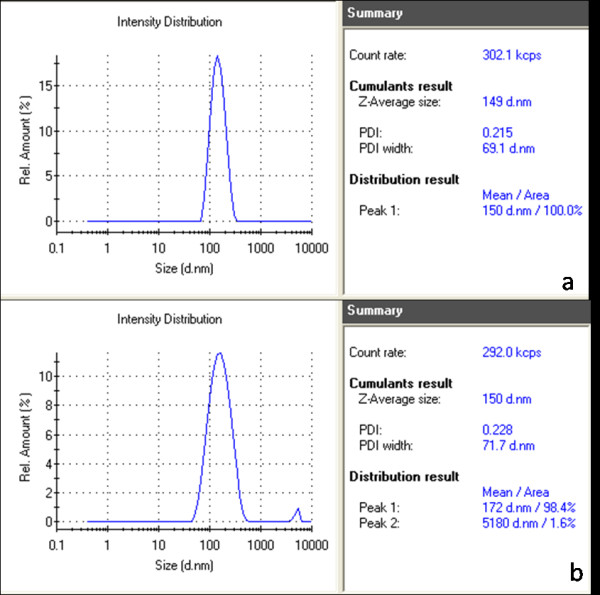
**Size distribution of HMSNs.** The size distribution of HMSNs was detected by Nano-sizer before(a) and after(b) protein loading.

HMSNs with protein complexation show slight increases in diameter (Figure 3b) compared with the HMSNs only (Figure 3a). The uniform size distribution of the HMSN/protein mixture at a diameter of about 172 nm suggests that the mixture is suspended well in solution. Another peak of size distribution is found at a diameter of about 5000 nm.

### Adsorption of protein

**Figure 4:**
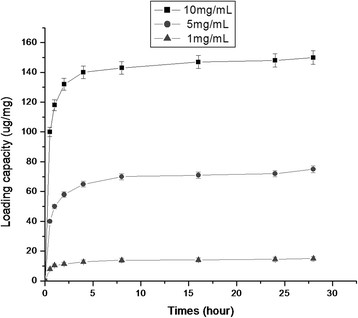
Adsorption kinetics of HMSNs for PCV2 GST-ORF2-E protein.

The amount of protein trapped within the HMSNs was determined by detecting the different concentrations of HMSNs in the supernatant before and after loading with the PCV2 GST-ORF2-E protein. Figure 4 shows that the loading of PCV2 GST-ORF2-E protein into the HMSNs is dependent on the solution concentration of HMSNs. The highest adsorption PCV2 GST-ORF2-E protein in the HMSNs is obtained at HMSN concentrations of 10 mg/mL. The maximum amount of loaded proteins is determined to be 150 μg per 1.0 mg of HMSNs in the present study. Taking these results into consideration, a nanoparticle concentration of mg/mL is selected for the optimal loading of PCV2 GST-ORF2-E proteins in all subsequent experiments. Absorption seemed to occur in a two-step pattern in all concentrations of HMSNs. Rapid absorbance of the protein is observed during the first 2 h of loading, followed by a second, relatively slow loading phase occurring in the next 30 h after.

### Release of protein

**Figure 5:**
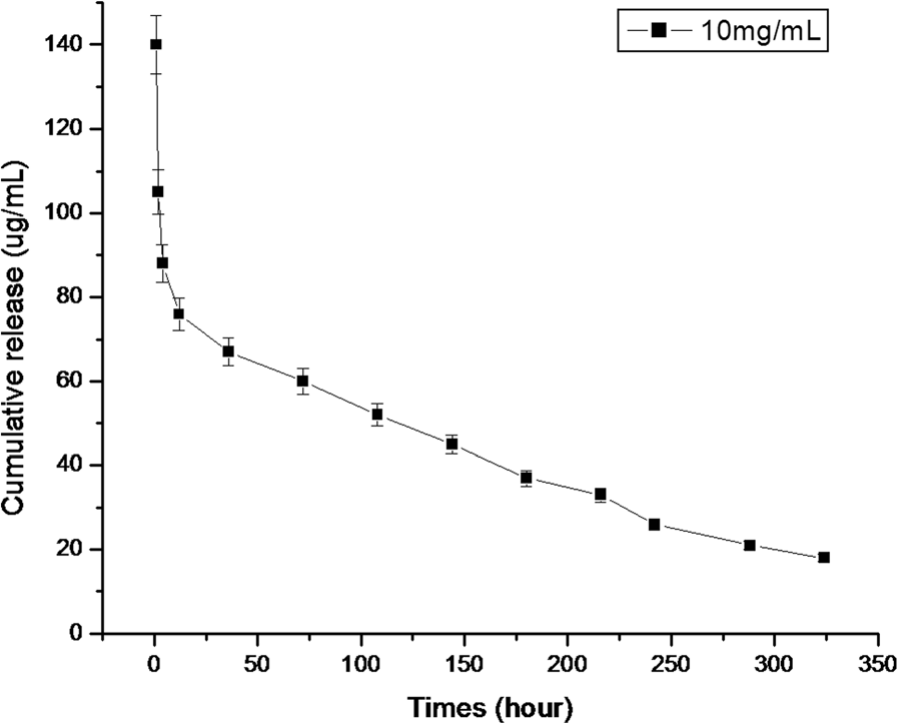
Cumulative release kinetics of PCV2 GST-ORF2-E protein from HMSNs in PBS at pH 7.0.

The release of PCV2 GST-ORF2-E protein from HMSNs (10 mg/mL) at room temperature was conducted in PBS (pH 7.0). Figure 5 shows the cumulative release kinetics of the PCV2 GST-ORF2-E protein. The release profile can be divided into two regions in a time-dependent process. A rapid release is observed up to 12 h after vaccination. During this time, about 50% of the encapsulated PCV2 GST-ORF2-E protein is released until the sixth day after immunization. A slower release is observed afterwards.

### The specific antibody of PCV2

**Figure 6:**
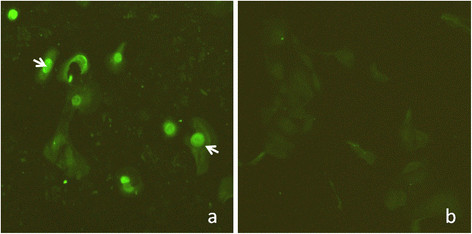
**Identification of specificity of mouse antibodies in PK15 cells infected with PCV2 by immunofluorescent microcopy.** (**a**) PK15 cells were infected with PCV2 at 10^-4.3^ TCID50 for 72 h, and then incubated with mouse antibody to PCV2 GST-ORF2-E. (b) Non-infected PK15 cells were used as a negative control.

To evaluate the specificity of mice antibodies immunized by GST-ORF2-E, mouse sera were used in direct immunofluorescence experiments to determine the specificity of antibodies by PCV2-infected PK15 cells. The specific fluorescence is located predominantly in the nucleus and, to a lesser extent, the cytoplasm of infected cells (Figure 6a). No significant staining was observed in mock-infected cells (Figure 6b), indicating the specificity of the mouse antibody against PCV2.

**Figure 7:**
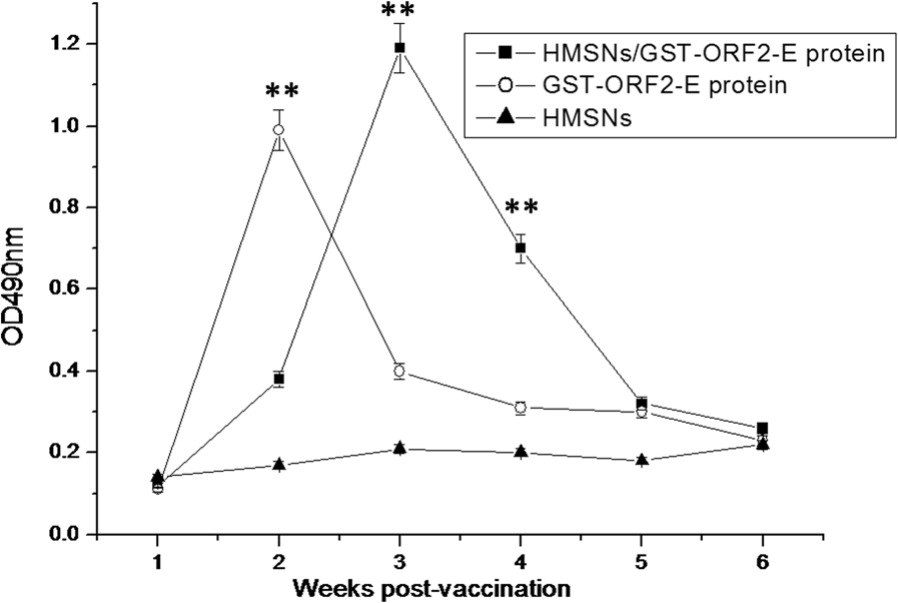
**PCV2 specific serum antibody responses.** Mice were immunized with the HMSN/protein complex, the protein only, or the HMSNs only;serum samples were collected every 2 weeks. Specific antibody responses in serum samples of 0, 14 and 28 d were detected by ELISA as described in Section 2. Each bar represents average values of three mice, measured in duplicate.

Indirect ELISA was performed to detect the titer of mouse-specific antibodies against PCV2 GST-ORF2-E protein. Figure 7 shows that the PCV2-specific antibody titers of mice vaccinated with the GST-ORF2-E protein greatly increase at the second week and decrease significantly at the third week post-vaccination. However, the antibody titers of mice immunized with the HMSN/protein mixture increase continuously, reaching a maximum at the third week post-vaccination. The antibody titers of mice then decreased gradually until the fifth week post-vaccination. The results demonstrate that the antibody titers of mice immunized with HMSNS/GST-ORF2-E are significant compared with those of groups immunized with GST-ORF2-E or the HMSNs at the third (p < 0.05) and fourth (p < 0.05) weeks after immunization. The antibody titers of mice immunized with GST-ORF2-E were statistically significant at the second week compared with those of the group immunized with HMSNs (p < 0.05).

### Lymphocytes proliferation assay

**Figure 8:**
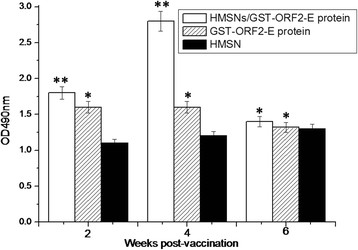
**T-cell responses elicited by immunization with HMSN/protein complex or protein or HMSNs.** Spleen cells were harvested at certain weeks post-immunization and restimulated in vitro with PCV2 GST-ORF2-E protein. Results show the mean ± SD of triplicate wells in each condition.

To measure T cell proliferative responses, splenocytes of mice were isolated and restimulated in vitro with purified PCV2 GST-ORF2-E protein. As shown in Figure 8, the proliferative capacity of the splenocytes is significant after immunization with HMSN/GST-ORF2-E at the second (p < 0.05) and fourth (p < 0.05) weeks compared with that of the group immunized with the HMSNs controls. Compared with group immunizaed with HMSNs, the T-lymphocyte proliferation in mice immunized with GST-ORF2-E is not significant at the second (p > 0.05) and fourth (p > 0.05) weeks post-immunization.

### T-lymphocytes subpopulations assay

**Figure 9:**
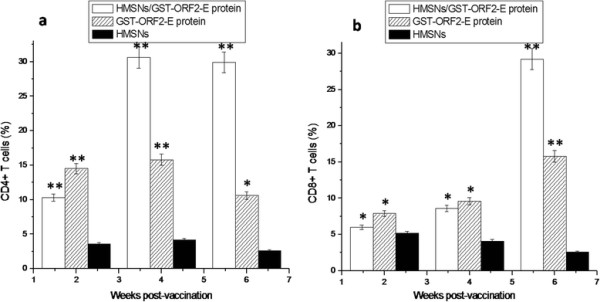
Percentage of the CD4+ and CD8+ cell population in spleen of mice immunized with HMSN/protein complex or protein or HMSNs.

The proportions of CD4+ and CD8+ splenocytes were determined by FCM. Figure 9a shows that CD4+ cells are elicited in the groups immunized with the HMSN/protein mixture and GST-ORF2-E at the second week but CD4+ cells of groups immunized with the HMSN/protein mixture upregulated significantly at the fourth week compared with that in the group injected with HMSNs only (p < 0.05). The proportions of CD4+ splenocytes remained high in mice of groups A at the sixth week post-vaccination. In contrast, the proportions of CD8+ cells in mice immunized with HMSNS/GST-ORF2-E or GST-ORF2-E proteins do not increase at the second and fourth week’s post-immunization (p > 0.05). The proportion of CD8+ cells in mice immunized with HMSN/GST-ORF2-E show significant increases at the sixth week (p < 0.05) (Figure 9b). In addition, the proportion of CD8+ cells in mice immunized with GST-ORF2-E proteins also increase at the sixth week, it is significant compared with that in mice immunizaed with HMSNs (p < 0.05).

### Mouse IFN-γ of serum

**Figure 10:**
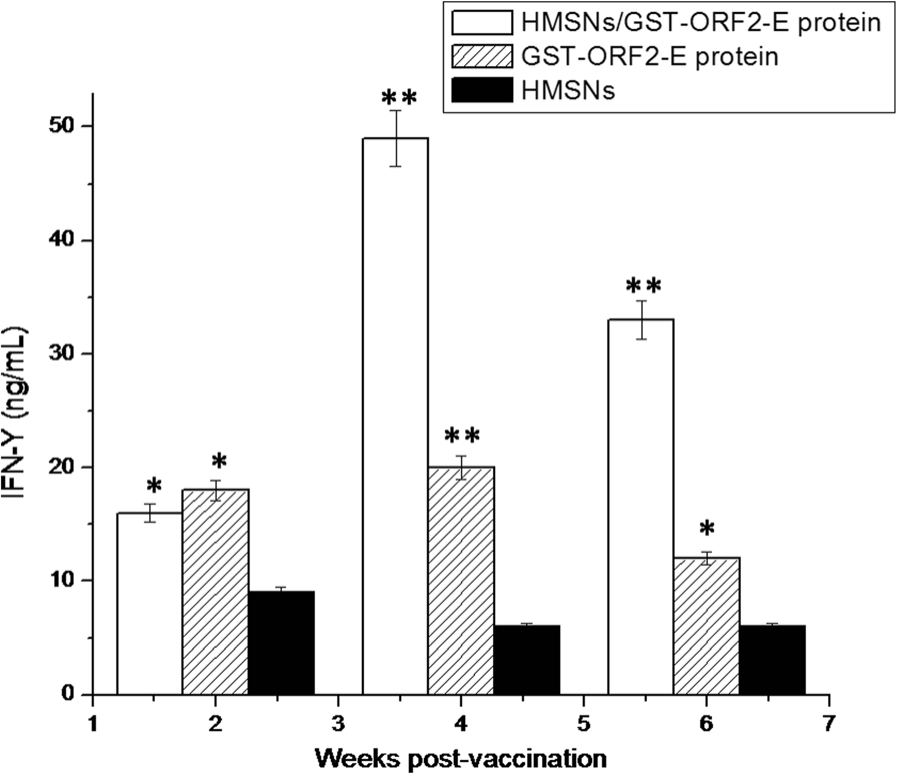
**IFN-γ production determined from the supernatant of splenocytes harvested from immunized mice.** Splenocytes were isolated 2, 4, and 6 weeks after immunization. IFN-γ production in the supernatant was analyzed by ELISPOT after stimulation with PCV2 GST-ORF2-E. Data represent the means ± S.D.

To determine the cytokine levels induced by the protein, an ELISA kit was used to measured levels of the Th1 cytokine, IFN-γ. Figure 10 shows that the levels of IFN-γ in the group immunized with HMSN/GST-ORF2-E are induced at the second week and increased significantly at the fourth (p < 0.05) and sixth (p < 0.05) weeks compared with those of the group immunized with HMSNs only. The levels of IFN-γ in the group immunized with GST-ORF2-E only increase significantly at the fourth weeks (p <0.05) and not remain at the sixth weeks compared with those of mice immunized with HMSNs.

## Discussion

In the literature, the ORF2 protein of PCV2 is a major candidate antigen for the development of vaccines and serological diagnostic methods. In the present study, the antigenic epitope of ORF2 was expressed and used as vaccine model to induce a specific immune response. Recent interest in mesoporous silica materials for use as carriers in controlled drug release has increased; such materials could meet the need for prolonged and better control of drug administration [23-25]. To induce persistent immune responses against specific antigens, HMSNs were used as protein carriers in the present study. Previous studies have reported the use of mice as a model for PCV2 infection [12,26]; thus, mice were immunized with ORF2-E protein-loaded HMSNs and their specific immune responses were determined.

Hollow mesoporous silica spheres were prepared by a sol–gel/emulsion (oil-in-water/ethanol) method, resulting in the formation of a hydrated silica gel on the surface of HMSNs. This surface layer had a mesoporous structure, with a pore diameter of about 2 nm [22]. Indeed, this unique structure provides the HMSN particles with a large surface area and pore volume, thereby enhancing their capacity for protein adsorption and allowing the hosting of chemical agents within them. This feature of HMSNs makes them very attractive options for use in material loading purposes. SEM images further demonstrate that the nanoparticles can be well dispersed in water, thus providing maximal interfaces with which to carry optimal amounts of chemical agents for delivery. Results obtained from the size distribution investigation reveal no clear difference in the conformation and size of HMSNs before and after adsorption of the protein. These findings confirm the stability of the HMSNs and further prove the suitability of the materials for use in carrier applications.

The present study reveals that HMSNs have a high adsorption capacity for GST-ORF2-E proteins at ratio of 150 ug/mg (protein: HMSNs), thereby decreasing the dosage of HMSNs in the HMSN/protein mixture and eliminating potential adverse effects resulting from high concentrations of HMSNs[22]. This advantage indirectly improves the immune responses of proteins loaded into HMSNs. Release kinetics findings showed that proteins adsorbed by the HMSNs could be released continuously over two weeks in PBS. The persistent release of proteins can provide a long-term stimulation to the immune system, which can be confirmed by persistent immune responses, including antibody titer and T-lymphocyte proliferation. To improve HMSNs and offer prolonged release kinetics, other possible factors related to the rate of release, including pore size and the payload capability of HMSNs, should be considered in future studies.

As expected, the antibody titers of mice immunized with the HMSN/GST-ORF2-E protein mixture were higher than those of mice immunized with the GST-ORF2-E protein, especially at the third and fourth weeks post-immunization. Furthermore, the T-lymphocyte proliferation response in mice induced by the HMSN/GST-ORF2-E protein mixture remained at levels higher than those in mice immunized with the GST-ORF2-E protein, demonstrating that the proteins loaded into the HMSNs not only stimulate humoral and cellular immune responses but also induce persistent immune responses because of the release kinetics of the HMSNs.

In literature, the percentage and ratio of two main lymphocyte T subsets, namely CD4+ cells or T helpers and CD8+ or cytotoxic T cells, have been recognized as the most meaningful parameters for evaluating the balanced state of immunomodulation and response to homeostasis of the intrinsic immune system [27]. CD4+ T cells have been proven to differentiate into two phenotypes: T helper type 1 (Th1) cells, which drive the immune response towards a cell-mediated immune response, and T helper type 2 (Th2) cells, which promote a humoral or allergic response [28]. CD8+ T cells are capable of inducing the death of infected somatic or tumor cells; they kill cells that are infected with viruses (or other pathogens), or are otherwise damaged or dysfunctional. Those cells that survive positive and negative selection differentiate into single-positive T cells (either CD4+ or CD8+), depending on whether their T-cell receptor (TCR) recognizes an MHC class I-presented antigen (CD8) or an MHC class II-presented antigen (CD4). CD4^+^ T cells have TcRs with an affinity for Class II MHC, and it is believed that CD4 is involved in determining MHC affinity during maturation in the thymus. CD8+ T-cells mature and go on to become cytotoxic T cells, following their activation with a class I-restricted antigen. The present study demonstrates that the HMSN/GST-ORF2-E mixture and GST-ORF2-E protein can elicit T-lymphocyte proliferative responses after immunization. Moreover, the percentage of CD4+ T or CD8+ T cells in mice immunized with HMSN/GST-ORF2-E is higher than that in mice immunized with HMSN and is higher than that hat in mice immunized with GST-ORF2-E protein at the fourth weeks and the sixth weeks. However, the percentage of CD4+ T cells in the spleen of mice immunized with HMSN/GST-ORF2-E is higher than that of CD8+ T cells at the fourth week post-vaccination. Six weeks post-vaccination, the percentage of CD8+ T cells increases to levels almost as same as that of CD8 + T cells. These findings suggest that CD4+ T cells can be stimulated prior to CD8+ T cells after immunization with HMSN/GST-ORF2-E, which will promote the proliferation of CD8+ T cells, and confirm previous conclusions that immunity associated with Th1 responses is essential for cytotoxic T lymphocyte production. The percentage of CD8+ T cells in the present study increased during subsequent weeks (the sixth week) after immunization. The continuous increase in CD8+ T cells provides protection against virus infections, an advantage of the PCV2 proteins released by HMSNs.

CD4+ Th1 cells mediate the killing of organisms responsible for a variety of intracellular infections through the production of IFN-γ[29,30], which induces the activation of macrophages and delayed-type hypersensitivity [31,32]. These cytokines can dramatically affect not only the strength of the immune response, but also its character [33]. In the present study, the Th1-associated cytokine IFN-γ was expressed after immunization with the HMSNs/GST-ORF2-E protein mixture, suggesting that a strong Th1 immune response was induced.

In conclusion, the present work highlights exciting research progress on the utilization of HMSNs as protein delivery agents. Further improvements in the mechanisms of the nanoparticles, such as increases in the capacity for protein absorption and consistency of release, may be expected in future work. While the results obtained are encouraging and show great potential for future applications, new breakthroughs and antigen delivery systems are still needed for this type of protein. The results confirm that HMSNs as vaccine carriers can improve cellular and humoral immune responses, especially persistent immune responses. Future studies could yield other exciting research accomplishments in the growing field of nano-vaccine-materials.

## Competing interests

The authors declare that they have no competing interests.

## Authors’ contributions

HCG and XMF designed the study and carried the synthesis of HMSN, and written the manuscript. SQS designed the research and revised the manuscript. YQW and DHS carried out the animal experiments. XTL and ZXL participated in the detection of immune response. JX L and HY participated in the statistical analysis. All authors read and approved the final manuscript.
